# Mesh Optimization for Monte Carlo-Based Optical Tomography

**DOI:** 10.3390/photonics2020375

**Published:** 2015-04-09

**Authors:** Andrew Edmans, Xavier Intes

**Affiliations:** Department of Biomedical Engineering, Rensselaer Polytechnic Institute, 110 8th street, Troy, NY 12180, USA

**Keywords:** Monte Carlo, mesh-based Monte Carlo, optical tomography, fluorescence molecular tomography, time-gated optical tomography, mesh optimization

## Abstract

Mesh-based Monte Carlo techniques for optical imaging allow for accurate modeling of light propagation in complex biological tissues. Recently, they have been developed within an efficient computational framework to be used as a forward model in optical tomography. However, commonly employed adaptive mesh discretization techniques have not yet been implemented for Monte Carlo based tomography. Herein, we propose a methodology to optimize the mesh discretization and analytically rescale the associated Jacobian based on the characteristics of the forward model. We demonstrate that this method maintains the accuracy of the forward model even in the case of temporal data sets while allowing for significant coarsening or refinement of the mesh.

## 1. Introduction

Fluorescence Molecular Tomography (FMT) is a highly sensitive molecular imaging modality which benefits from the availability of numerous molecular probes and relatively low cost. FMT is especially valuable in preclinical studies as it allows for three-dimensional, quantified reconstructions of the distribution of fluorescence probes in tissue. This has numerous applications in fields such as drug discovery and gene therapy [1–3]. FMT is based on an inverse problem, which links a measurement vector and a forward model of light propagation to successfully retrieve the unknown parameter distribution in the image space. For simplicity of implementation, the diffusion equation (DE), an approximation of the radiative transport equation (RTE), is usually used as the forward model. Typically, DE-based algorithms are two orders of magnitude faster than RTE-based ones, with the added advantage of being more robust [4–6]. However, the DE approximation does not accurately represent the propagation of photons when absorption is large compared to scattering, when scattering is low, near boundaries and for ballistic or minimally scattered photons [7–10]. Hence, there are still considerable interests in developing efficient RTE-based algorithms to overcome these limitations, which are often encountered in pre-clinical imaging applications. Recently, the Monte Carlo (MC) method, which is a stochastic solver to the RTE [11], has been proposed as an accurate and general forward model for optical tomography [12,13].

The Monte Carlo (MC) method simulates the path of numerous photons through complex tissue to sample the imaged volume with suitable statistical accuracy [14]. This method is considered the gold standard for photon transport modeling in bio-tissues and is frequently employed as a benchmark when developing novel optical techniques and algorithms [15]. Especially in time resolved imaging, MC techniques provide improved accuracy in forward models compared to common alternatives such as the diffusion equation, as it can simulate both minimally scattered and diffuse photons [16]. However, MC techniques were not considered viable to generate the forward model in optical tomography due to the necessity of computing numerous forward simulations, with each simulation potentially requiring hours of computing time [17,18]. Recently, thanks to new MC formulations [19,20] and massively parallel implementations [21,22], the computational cost of MC simulations has drastically reduced. For instance, our group first demonstrated that functional and fluorescence optical tomography is feasible within frameworks that are competitive with DE-based algorithms [14,23]. Especially, MC techniques based on finite element mesh models (mMC) have been developed which lessen this computational burden and also improve the modeling of boundary conditions for complex geometric models [24,25]. mMC, along with parallel implementations [26], allow for Monte Carlo based fluorescence molecular tomography to be implemented with reasonable time and computation demands in the case of free space imaging [27], challenging data types such as early gates [28], and wide-field strategies [23,29,30]. However, mMC does not yet benefit from adaptive finite element methods that are frequently employed in PDE based inverse problems [31].

For arbitrary domain geometry, the FMT problem poses a tradeoff between accuracy and computational efficiency [32]. As FMT is typically highly underdetermined, the choice of uniform nodal spacing results in higher computational complexity compared to meshes that have nonhomogeneous nodal spacing [33]. Hence, adaptive mesh discretization techniques have been implemented for diffuse optical tomography applications. To date, these mesh optimization strategies are limited to analytical-based light propagation models. Especially, adaptive meshing algorithms have been developed for DE-based tomographic image reconstruction procedures [34–37], where the emphasis is on refining a coarse mesh at the region of the heterogeneity(ies). These adaptive mesh refinement techniques not only improve localization and quantification of sources [38,39] but also enhance the robustness and efficiency of reconstructions [40,41], though at an additional computational cost. In all adaptive mesh approaches, the Jacobian needs to be fully constructed at each iteration, leading to a significant additional computational cost, typically ~75% of the computation time, at any given iteration [42]. This is a critical issue when attempting to apply these techniques to MC based approach.

Indeed, Monte Carlo methods require simulating the propagation of a large number of photons (10^6^–10^9^) per simulated optode, depending on the data type, in preclinical settings [14]. As the accuracy of the MC-based Jacobian is dependent on the local statistics of the forward problem, coarsening or refining the mesh requires recomputing the Jacobian, possibly with a greater photon packet number to satisfy smaller discretizations. The iterative nature of adaptive mesh techniques may then lead to hours of computations, even in a massively parallel environment. Herein, we investigate the application of fast and efficient forward mesh optimization approaches for time resolved MC-based FMT. We propose a mesh optimization methodology in which the initial mesh and MC forward models are analytically rescaled at each iteration, allowing for fast computation without loss of accuracy.

## 2. Methods

### 2.1. Optical Inverse Problem

The goal of FMT is to retrieve the 3-D distribution of a fluorophore typically expressed as its effective quantum yield: *η*(*r*) [13]. This distribution can be obtained by solving the integral equation at time *t*:

(1) $$U_{F}(r_{s},r_{d},t)=\int_{\Omega }W(r_{s},r_{d},r,t)\eta (r)dr$$

where *U_F_*(*r_s_*, *r_d_*, *t*) is the fluorescence detected at the detector *r_d_* at time *t* resulting from the initial excitation from the propagation or Jacobian. To efficiently calculate the Jacobian, *W*, based on the mMC forward model computation, we employed the forward-adjoint method [14]. In this formulation, *W* is computed by convolving the Green functions and the fluorophore lifetime decay:

(2) $$W(r_{s},r_{d},r,t)=\int_{0}^{t}e^{-(t-t^{\prime })/\tau }dt^{\prime }\int_{0}^{t^{\prime }}G^{x}(r_{s},t,t^{\prime }-t^{\prime \prime })*G^{m}(r,r_{d},t^{\prime \prime })dt^{\prime \prime }$$

where *G^x^* and *G^m^* are time-dependent Green’s functions (the light propagation for impulse sources) calculated by mMC and *τ* is the lifetime of the fluorophore. The weight matrix and detected measurements at different positions can then be represented as a system of linear equations and solved to obtain the fluorophore biodistribution.

### 2.2. Mesh-Based Monte Carlo

The details of the mesh Monte Carlo method used here can be found in reference [24]. In brief, the mMC method utilizes fast ray-tracing to accelerate calculation. Our implementation allows simulation of arbitrary sources illuminating complex geometries with very small computational overhead compared to point source illumination (5%–10% increase) [26]. The method has been implemented using single-instruction multiple-data (SIMD) and Message Passing Interface (MPI) to allow for both multithreading and multiple node computation within an adaptive hybrid parallelization architecture. We reported that this mMC implementation is 5× faster than its voxel based counterparts, with the benefit of producing more accurate simulations in the case of free space pre-clinical imaging [26].

### 2.3. Mesh Adaptation

**Figure 1:**
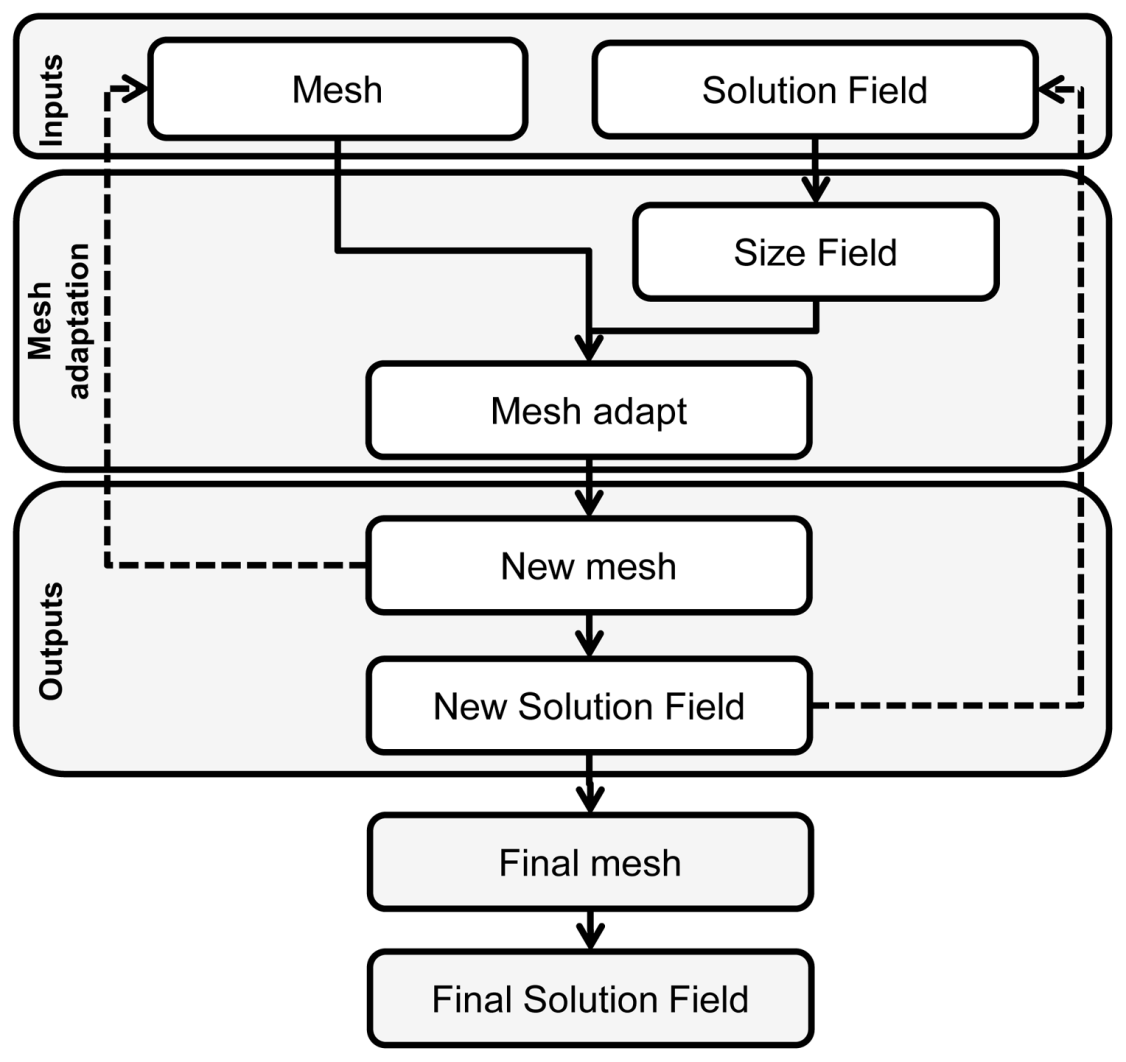
Flowchart of the mesh iterative adaptation program.

Mesh adaptation was achieved using the method laid out in [43] and implemented in the MeshSim Adapt package (Simmetrix, Inc, Clifton Park, NY, USA). This mesh adapt package allows for iteratively adapting a finite element mesh to fit an input size field that consists of a size factor at each node of the finite element mesh. The size factor associated to each node dictates the local mesh adaptation operation that will occur at this iteration. A flowchart of the overall optimization process is summarized in Figure 1.

Size factors greater than one lead to mesh coarsening whereas size factors lower than one lead to mesh refining. This size field is created from a solution field that is relevant to the formulation of the FMT inverse problem. To convert the solution field to a size field, each value of the solution field is compared to two thresholds: a lower one and an upper one. If the solution value is lower than the lower threshold, then the size factor at that node is equal to a set maximum size factor. If greater than the upper threshold, then the size factor is equal to a set minimum size factor. Otherwise, when between the two thresholds, the solution field value is converted to a size factor *y* following:

(3) $$y={pa}^{x};\;a={\left(\frac{1}{1.25}\right)}^{\left(1/1.4*\text{Median}\right)};\;p=\frac{1}{1.6*\text{Median}}$$

where *x* is the solution field value and *y* is the resulting size factor. The foundation of the mesh adapt code was laid in [43].

**Figure 2:**
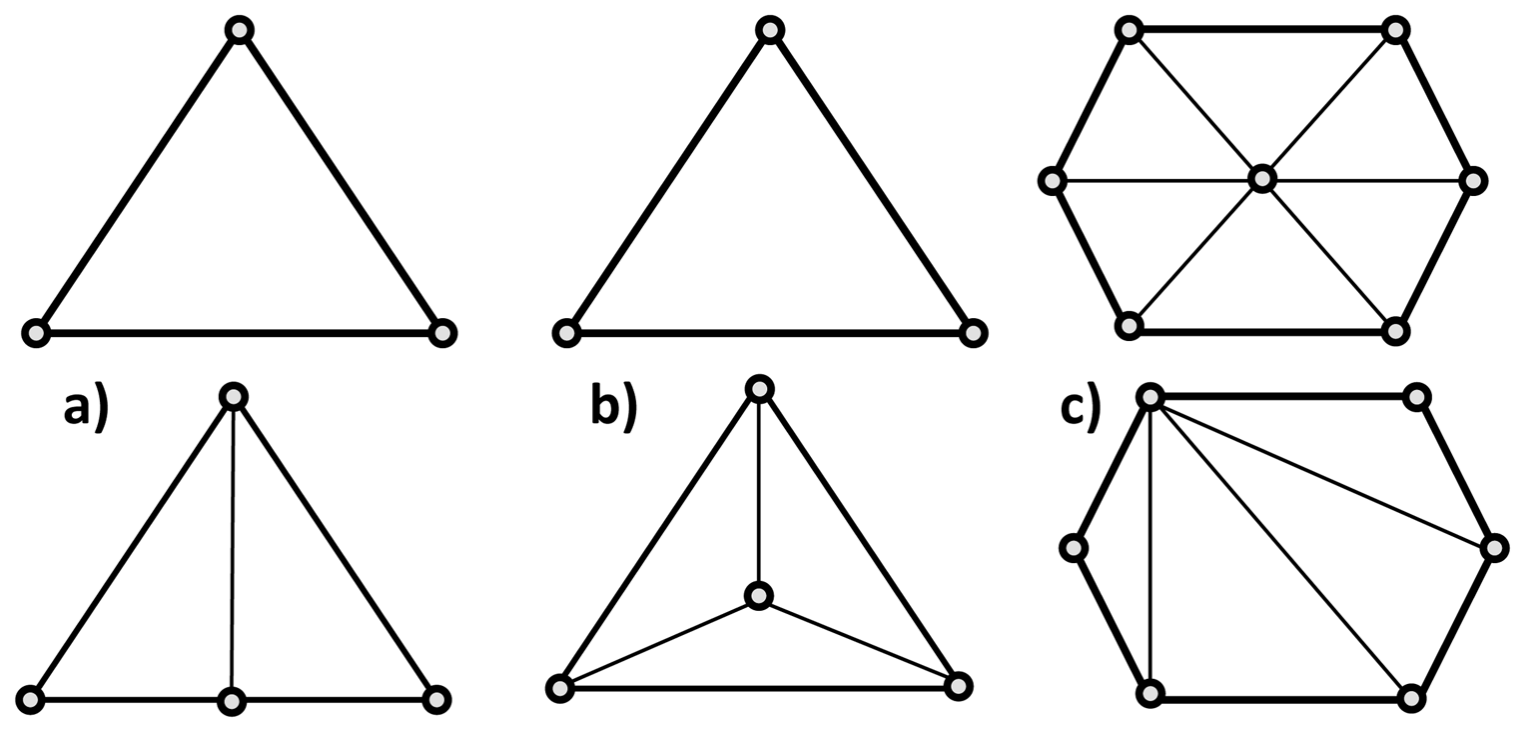
Examples of an (**a**) edge split, (**b**) face split, and (**c**) edge collapse.

The two main families of operations used to achieve adaptations are either split or collapse operations. Split operations introduce a new node to fragment an element of volume towards mesh refinement. A new node may be placed in the center of an edge, a face, or a region. New edges are then drawn accordingly. Thus, the number of nodes and elements are increased while also shortening the average attached edge lengths in the region. Figure 2a, b show an example of edge and face split in two dimensions. Vice versa, collapse operations are used to coarsen the mesh. Two types of collapses may be employed: edge and region. If an edge is selected for collapsing, one node of the edge is “pulled” towards the other node, eliminating one node and creating less elements in the area, as shown in Figure 2c. In region collapses, one, two, or three faces bounding the region to collapse are removed to combine elements.

The mesh adaptation is an iterative process in which the new mesh and a new solution field are used as inputs for the next iteration step. The process can be repeated until set stopping criteria (*i.e.* convergence in the number of elements/nodes, absolute number of elements/nodes, set element of volume attained, *etc.*) are met yielding an optimized mesh. However, at each iteration of the mesh adaptation, new mMC simulations should be carried out to reflect the changes in the discretization and hence nodal value of the Jacobian. This can become a burdensome process as, conversely to PDE-based problems, mMC computational burden is related to discretization level. Especially, in the case of mesh refinement, one can expect that increased number of photons is required to reach statistical stability to compute adequate Jacobians. Instead, we propose a technique to analytically rescale the forward model at each iteration in order to fit the new FEM mesh.

### 2.4. Jacobian Rescaling

The Jacobian is a system matrix *A* that describes the relation between the fluorophore effective quantum yield in every 3D image element (*e*_1_, *e*_2_, …, *e_n_*) and measurement data set. During the mesh adaptation procedure, the number and location of the image element may change and thus, the Jacobian needs to be recomputed to maintain accuracy. Ideally, the Jacobian would be recomputed using mMC, but at a significant computational cost. Another approach is to identify, by comparing the old mesh and the new mesh, the nodes that have been modified and extrapolate analytically the value of the Jacobian for these nodes without new stochastic computations. For this, a transformation matrix is generated that links the input to the output mesh where each column represents a node in the output mesh and each row represents a node in the input mesh. The entries of the matrix describe the changes between the input and output meshes. Therefore, this matrix is used as a transformation operation which provides an accurate Jacobian matrix for the modified mesh by multiplying the input mesh by this transformation matrix.

**Figure 3:**
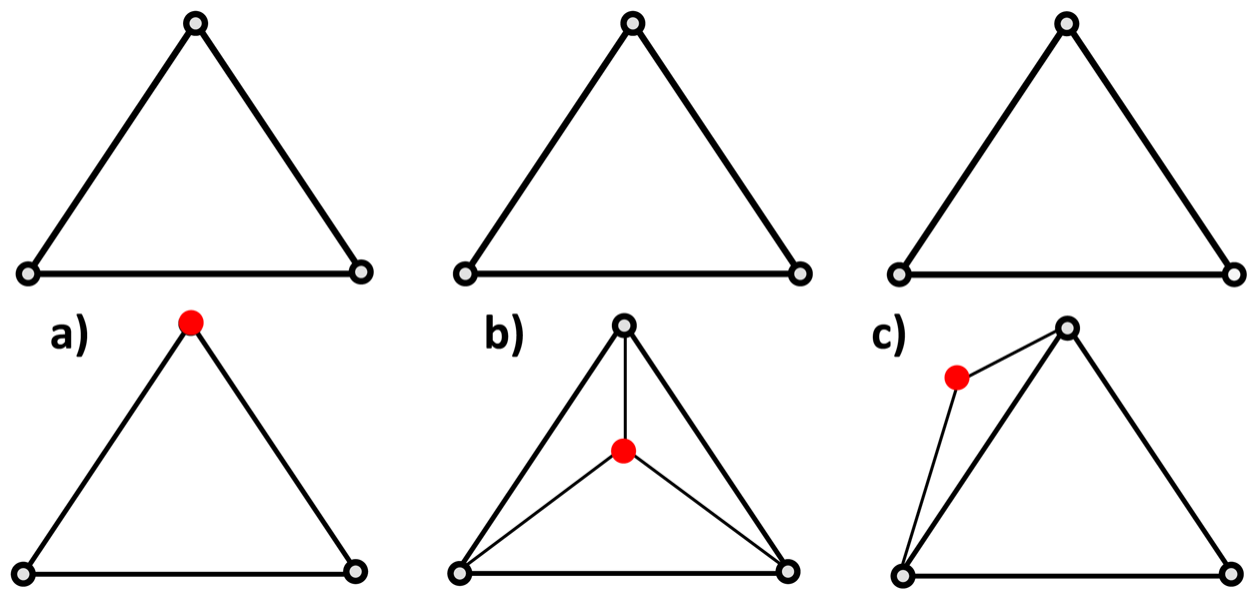
The possible cases in the output node positioning. The upper row corresponds to the input discretization and lower row to the output mesh.

As each output node is compared to the input nodes, three conditions may occur. First, this node has not been modified during the adaptation process. The Jacobian value is simply carried over to this node in the new mesh (Figure 3a). Second, the output node does not correspond to any input node and falls within a volume element in the input mesh (Figure 3b). In this case, a new Jacobian value is extrapolated based on the Jacobian value of the surrounding nodes in the input mesh. Specifically, the nodes of the input mesh which make up the enclosing element are weighted inversely to distance and summed to create the new nodal value. The coefficients used to weight the Jacobian nodal values for each new node *i* are calculated as:

(4) $$c_{i}=\frac{w_{i}}{\sum_{j=1}^{n}w_{j}}$$

where *n* is the number of nodes in the enclosing element and:

(5) $$w_{i}=\frac{1}{d(x,x_{i})}$$

where *d*(*x*, *x_i_*) is the distance from the new node *x* and the *i^th^* enclosing node. Each weight is placed in the column of the output mesh node in each of the rows corresponding to the enclosing nodes. Lastly, the new node may be outside the bounded region defined by the input mesh (Figure 3c). In this case, the same process as case two is carried out, except that the four closest nodes of the input mesh are found since there is no enclosing element. Once the transformation matrix is fully populated, the new Jacobian is computed by matrix multiplication of the input Jacobian with the transformation matrix.

### 2.5. In Silico Model

**Figure 4:**
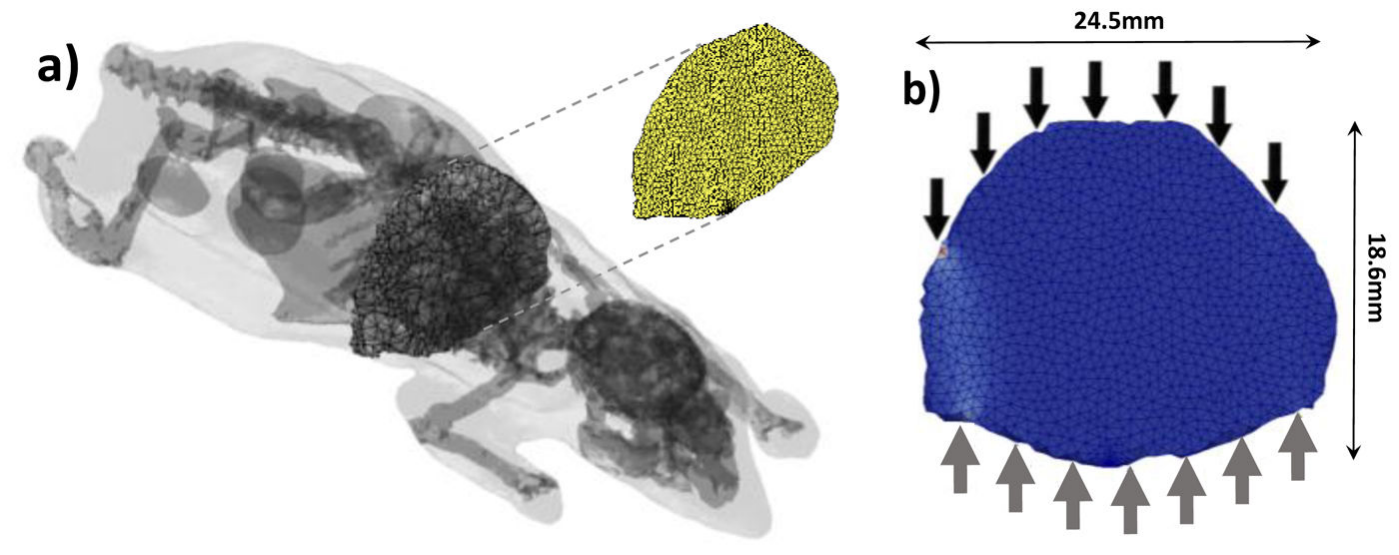
(**a**) The digimouse model with slice highlighted next to (**b**) the mesh section used with positions of sources (solid black) and detectors (solid grey). The slice is 4 mm thick.

The 3D digimouse model was employed to create an anatomically accurate *in silico* preclinical model. The average optical properties of the mouse were used to compute the forward model These optical properties were: *μ_a_* = 0.3 *cm*^−1^, $\mu_{s}^{\prime }=15\;{cm}^{-1}$, *g* = 0.9, and *n* = 1.37, which are typical of mouse tissues in the NIR spectrum [5]. The optical properties were considered homogenous over the animal as it is typically done in optical tomography. This simplification works well when using the Born/Rytov approximation experimentally in DOT, and the normalized Born formulation in FMT. Moreover, the optical properties of the different organs in live animals are still unknown to date. The Henyey-Greenstein phase function was employed to define the new photon direction after each scattering event. Overall, the 3D digimouse mesh contains 210,161 elements and 42,301 nodes. A 3D slice of the digimouse model containing 2396 nodes and 7574 elements is used as the initial mesh, as shown in Figure 4a (cross section of the torso). Then, 7 time-resolved point sources and 7 point detectors in transmission geometry were simulated as depicted in Figure 4b. This simplified model allows investigation of the impact of the size factor and solution field on computational efficiency of adaption mesh.

## 3. Results and Discussion

Mesh adaptation in the forward model space is expected to improve the computational efficiency of the Monte Carlo simulations without sacrificing accuracy in the forward model or reconstruction. The computational cost of mMC is mainly associated with the number of photons that need to be launched to reach statistical stability. Among the parameters that dictate the number of photons required, the level of discretization plays a critical role. As element volumes diminish, less photons sample these elements, leading to poor statistics. Moreover, in the case of preclinical imaging in which transmission geometry is used for optimal tomography performances, as the element of volume farther from the source are less likely to be visited by photons. Even if this issue can be mitigated with adjoint methods [20] and specialized filtering techniques [44], it is still necessary when employing homogeneous meshes to use photon packets that provide good statistics for the less visited elements (typically mid-plane between source and detector). With mesh adaptation, the elements that receive the least photons can be increased in size in order to yield better statistics. Hence, fewer photons can be used in later Monte Carlo simulations to achieve statistics similar to higher photon counts in less time, as computational time is linearly related to the number of photons simulated.

### 3.1. Size Factors

The mesh adaptation process is dependent on a few parameters which are set *a priori*, as described in Section 2.3. Especially, the maximum and minimum size factors greatly impact the convergence rate of the adaptation process as they bound the allowed range of changes in the elements of volume. Herein, we investigate the impact of the maximum size factor on mesh coarsening for mesh adaptation based on the forward model. As the application sought is to improve computational efficiency, no refinement is considered, and thus, the minimum size factor is set to 1.

To establish the best maximum size factor for our application, mesh adaptations with various maximum size factor values were conducted until they reached convergence using the cross sectional mesh configuration described in Section 2.4. Convergence is defined as the point when the output mesh of an iteration has the same number of nodes and elements as the input mesh for that iteration. The solution field as obtained by the sum of the Jacobian (see Section 3.3) was used with a lower threshold set to 20% of the median and an upper threshold 8 times the lower. Table 1 summarizes the mesh characteristics of each of the resulting convergence points compared to the initial mesh for three selected maximum size factors.

**Figure 5:**
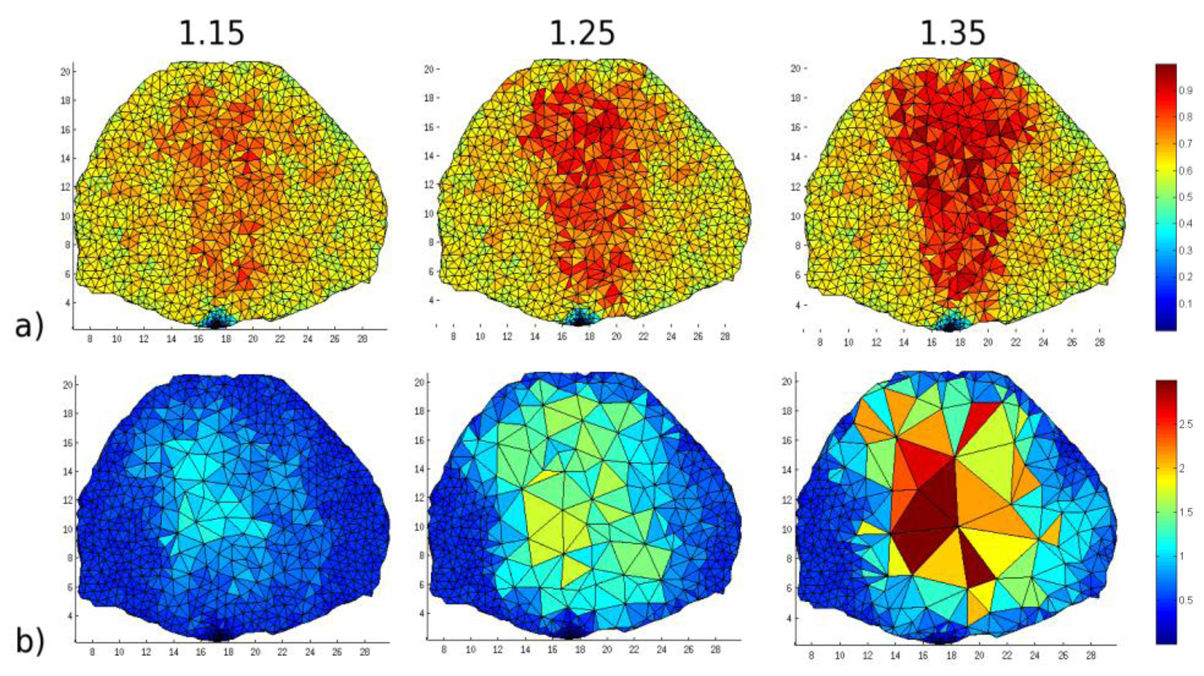
The mesh under different maximum size factors after (**a**) one iteration and (**b**) seventeen iterations, the greatest iteration all meshes achieve before convergence. Maximum size factors are provided as subtitles (color bar corresponds to volume of elements in mm^3^).

As the maximum size factors increase, the mesh coarsening is more pronounced, as expected. The coarsening leads to a maximum (minimum) reduction in the number of nodes by ×4 (×2) and in the number of elements, by almost ×5 (×2), compared to the initial mesh. The maximum element volume is almost 6 times larger for a maximum size factor of 1.35 compared to 1.15. However, the element of volume sizes are difficult to predict as the coarsening is mainly based on the edge lengths. For instance, in the case of the size factor of 1.35, the change in maximum element size from one iteration to another can be as high as 125% and as low as 12%. Hence, the convergence rate is not as stable as for smaller size factors. Additionally, for the smallest size factor (1.15), even if convergence is stable and achieved at an earlier iteration number, the coarsening is limited. Hence, a maximum size factor of 1.25 is considered to be optimal as it provides stable convergence and significant reduction in the mesh elements (×3) and nodes (×3.5). Figure 5 provides a visualization of the mesh after adaptation at the first iteration and the seventeenth.

### 3.2. Forward Model-Based Solution Fields

The solution field is the input to derive the size field, and hence, the field which determines the areas of the mesh that should be adapted based on the solution values at each node. Herein, the solution is computed based on the forward model, *i.e.*, the mMC simulations. For FMT purposes, the mMC simulations are used to compute the Jacobian. However the Jacobian is constructed based on numerous mMC forward simulations computed on the same mesh. Hence, for mesh adaptation purposes, it is required to obtain a solution field that represents the overall sensitivity of the forward model to the volume imaged, not to a specific source-detector pair. Herein, we consider five methods to derive a solution field based on the mMC Jacobian. First, we consider the most obvious solution field which consists of the sum of the rows of the Jacobian (Σ*Jacobian*). This operation yields a solution field with the value at each node representing the sum of all source-detector pair sensitivities at this node. Second, we consider the logarithm of the sum of all rows (*log*(Σ*Jacobian*)). As light propagation in highly scattering media is characterized by an exponentially overdamped scalar wave, the dynamical range in the forward model is very large. Using a logarithmic scale allows for mitigation of such high dynamical range and provides a more linearly distributed solution field. We tested also a curvature metric derived from a Laplacian-type operator as described in reference [45]. This metric (*u*) is defined as:

(6) $$u=LS;\mathrm{with}\;L=\left\{ \begin{array}{ll} 1, & if\;i=j\\ \frac{-1}{n-1}, & \mathrm{otherwise} \end{array}\right.$$

where *S* is the sum of the rows of the Jacobian and where *n* is the number of nodes in the mesh. Similar to the sum of Σ*Jacobian*, we tested also the *Log*(*u*). Last, we considered the sum of the rows after each row has been normalized to the same maximum value (*normalized* Σ*J*). As the boundary conditions of the model are not planar, the different source-detector pairs are visiting volumes of different thicknesses. Normalizing each row of the Jacobian allows for mitigation of the dynamical range associated with the curved boundaries to yield a more homogenous solution field.

Once the solution field is obtained, a size field is produced to be used as an input for the mesh adaptation procedure. As mentioned above, the size field is computed based on the median value of the solution field and set thresholds. Here, the lower threshold was set to 20% of the median and the upper threshold to 8 times the lower. Under the varying solution fields, each mesh is brought to a unique convergence point as with the size factors. The characteristics of the optimized meshes at convergence are provided in Table 2.

Overall, the sum of Σ*Jacobian* and curvature fields (*u*) provide the most reduction in mesh elements/nodes. Both, *log*(Σ*Jacobian*) and *normalized*Σ*J* leads to coarsening but converge to maximum elements of volume that are still relatively small compared to Σ*Jacobian*. Hence, these two solution fields are less attractive than the Σ*Jacobian* and *u* metric. To further establish the merit of each solution field, a stopping criteria is implemented to stop the iterative adaptation when a set maximum element of volume is achieved. For real applications, this element of volume would ideally be close to the FMT system resolution or application-required resolution, to balance computational efficiency with image reconstruction accuracy. Here, we set the cutoff at 0.25 mm^3^, the smallest maximum volumes achieved at convergence. This allows for comparison of all solution fields as summarized in Table 3.

**Figure 6:**
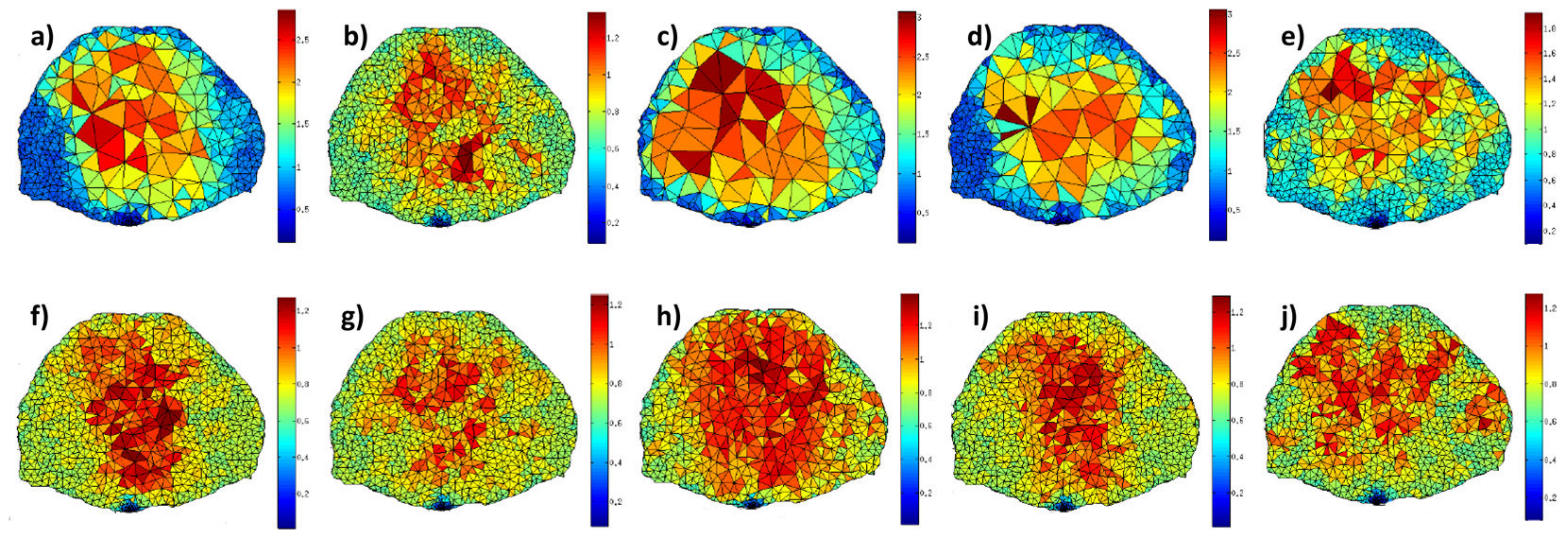
Upper row: mesh at convergence (**a**) Σ*Jacobian*, (**b**) *log*(Σ*Jacobian*), (**c**) *normalized* Σ*J*; (**d**) *u* and (**e**) *Log*(*u*); lower row: mesh at 0.25 mm^3^ stopping criterion for the respective corresponding input fields.

In this case, the curvature and Σ*Jacobian* are still the solution fields that lead to the fastest adaptation calculation whereas, the *normalized* Σ*J* leads to the most reduction in elements and nodes, but only ~10% less than Σ*Jacobian*. However, after only 2 iterations, the *u* field led to larger elements of volume than the stopping criterion (26.5%). Additionally, even if the *Log*(*u*) metric provides similar outcome than the Σ*Jacobian*, it is more difficult to implement. Using *Log*(*u*) (or *log*(Σ*Jacobian*)) leads to negative values in the input solution field, leading potentially to a negative Median in Equation (3). This affects the calculation of the size factors as it is possible for a node to have a value which is greater than the upper threshold and lower than the lower threshold, leading to instability during the iteration process. Thus the Σ*Jacobian* is selected as the most appropriate solution among the investigated model-based fields for preclinical FMT applications. Figure 6 provides the different meshes for all cases investigated.

### 3.3. Geometry-Based Solution Fields

**Figure 7:**
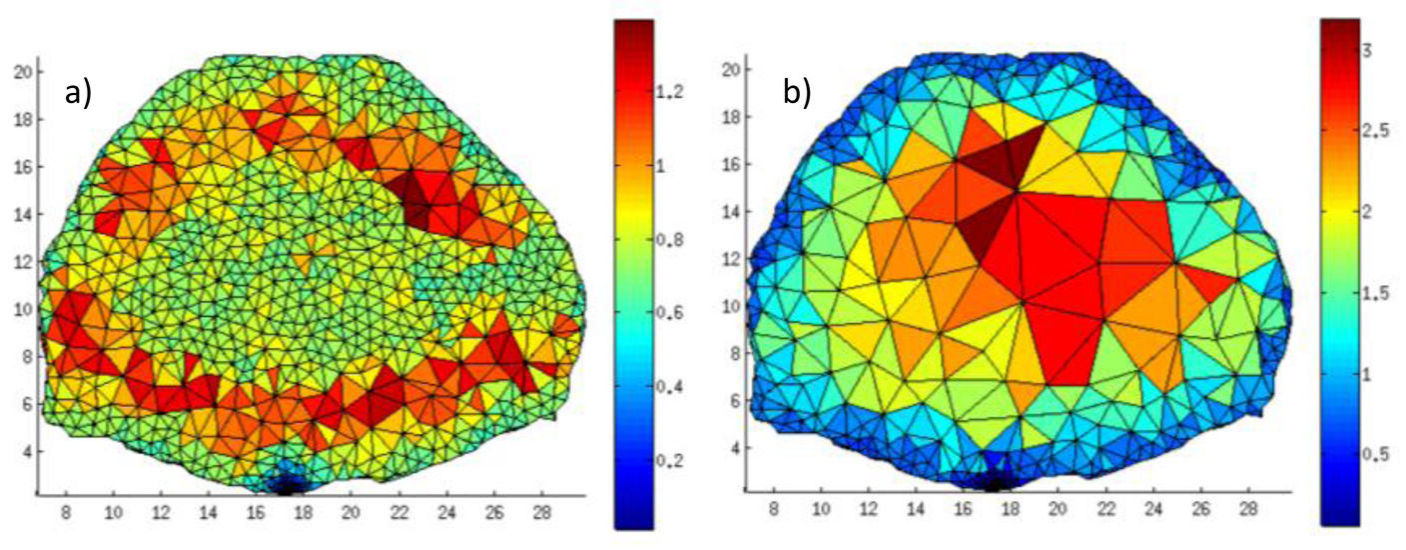
Mesh at convergence for (**a**) *Dist.* and (**b**) *Att*.

The solution fields which have been investigated up to this point require a pre-computed Jacobian to start the adaptation process. This can render the whole process computationally demanding. Another option for creating the solution field is to use the *a priori* geometrical information of the volume to image and optode locations to generate analytically the solution field. Keeping in mind that the goal of adaptation in the forward model is to optimize the stochastic stability of the MC forward model at every element of volume, the scaling field can be generated using the distance from these elements of volume to the optode location. Here, we investigate solution fields generated by two geometry-based analytical scaling methods. First, we compute the distance for every source and detector to each node and identify the minimum distance. This minimum distance at each node is our solution field. For this option, the size factor information is reversed because now the greatest values should be coarsened the most instead of the lowest values. We termed this approach *Distance Field* (*Dist.*). The second approach consists of weighting the minimum distance by an attenuation coefficient which is related to the optical properties of the medium. The attenuation coefficient follows a simple Beer-Lambert Law such that:

(7) $$\mathrm{Att}.=e^{-\mu x}$$

where *Att.* is the resulting solution field value, *x* is the distance to the nearest source/detector and *μ* is the total attenuation coefficient (absorption and scattering attenuation). We refer to this solution field as *Attenuation Field* (*Att.*). For this field, the lowest values are coarsened the most. Table 4 summarizes the mesh characteristics for each cases and side by side comparison is provided in Figure 7.

At convergence, the *Att.* field is comparable to Σ*Jacobian*, though the source *Dist.* is self-limited similarly to *log*(Σ*Jacobian*). This is an expected relationship as attenuation is based on an exponential and is similar to the sum. At convergence, the attenuation field mesh had only 3.8% fewer nodes with a maximum volume 3.1% larger than the Jacobian-based field. The *Att.* based optimization can be carried out in a few seconds thanks to its simplicity whereas the Σ*Jacobian* requires an initial MC forward computation that is significantly more time consuming. Hence, the *Att.* field approach is a very attractive analytical approach for optimizing the mesh in mMC applications, either as a method to generate an initial mesh or adapt the mesh for improved computational efficiency.

### 3.4. Jacobian Accuracy and Computational Efficiency

**Figure 8:**
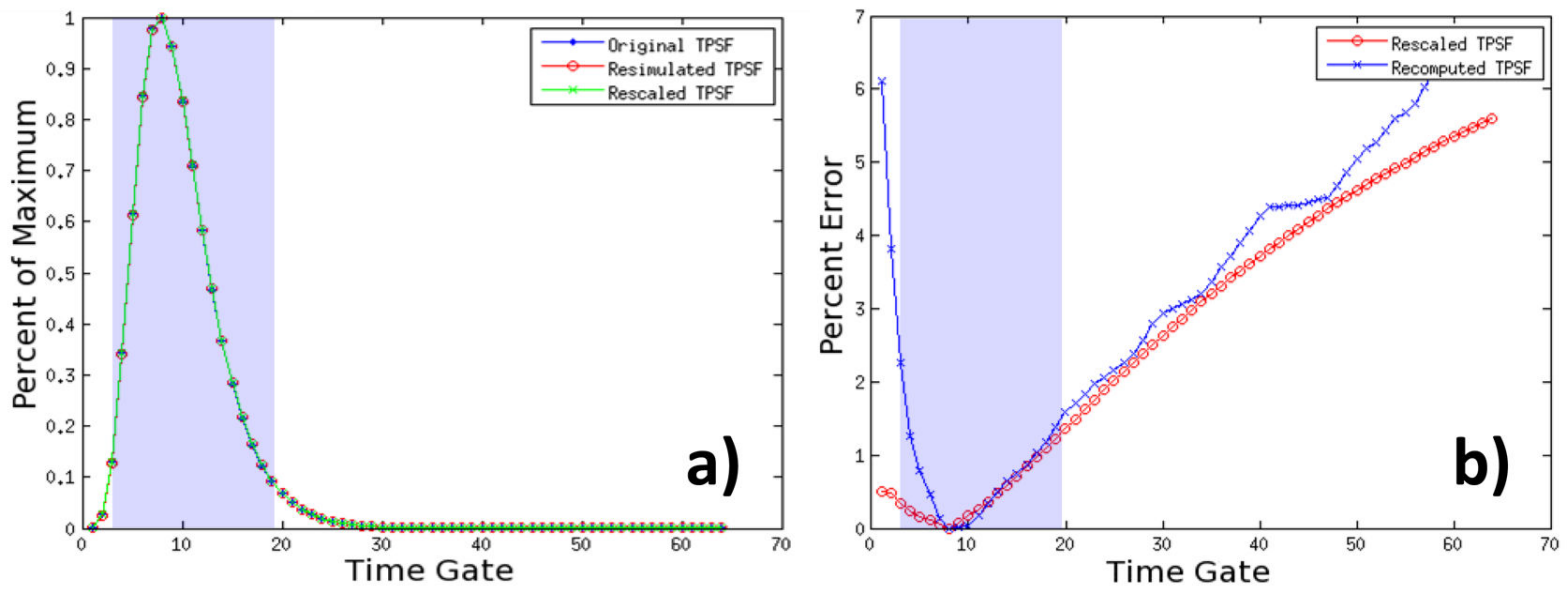
Temporal Point Spread Function (TPSF) of Jacobians rescaled to a new mesh and the associated error at each gate.

MC based FMT reconstructions are used when the DE fails to adequately model light propagation. As in the proposed method, an analytical rescaling is performed locally to adjust the mMC Jacobian to the new discretization. It is crucial to assess if model accuracy is maintained. To do so, we consider time resolved data types for which the early part is known to be modeled poorly by the DE. A Temporal Point Spread Function (TPSF) was computed from the mMC based Jacobian (10^9^ photons) prior to adaptation and from the analytically rescaled Jacobian after adaptation (10^9^ photons). Moreover, new mMC simulations were computed to obtain a mMC Jacobian on the new adapted mesh. An example of TPSFs produced for one specific source detector pair is provided in Figure 8a) (central pair). The TPSFs simulated under the original and mesh adaptation conditions are matching remarkably. Furthermore, the error in the TPSF from the rescaled Jacobian is similar to the error in the TPSF from the recomputed Jacobian. Throughout all time gates that are typically employed to cast the inverse problem, the maximum error is less than 1.5% for late gates and below 0.5% for early gates (Figure 8b). This error level is similar for all 49 simulated optode combinations. These results indicate that the mesh adaptation and analytical rescaling methodology described above lead to optimized non-homogenous discretization that does not affect light propagation accuracy, even in the most challenging cases, *i.e.*, early gates.

**Figure 9:**
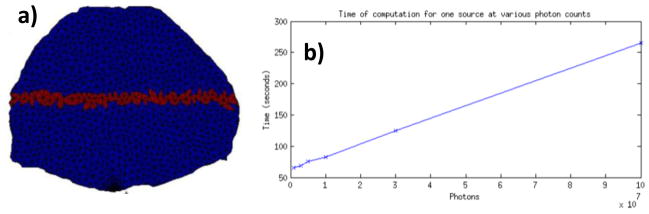
(**a**) Elements of volume employed to compute the error *e*(*r*) (red label); (**b**) Computation time for one forward simulation *versus* number of photons.

The goal of the mesh adaptation described herein is to conserve the stochastic reliability of the forward model while decreasing the computational burden. If more photons are reaching the center elements (ones with poor statistics in the adjoint method), lower photon packets can be simulated for each optode. The relationship of computational time against the number of photons simulated is linear, as shown in Figure 9b, in our mMC implementation. To assess the impact of mesh optimization as described above on the Jacobian stochastic stability, we computed the Error (*e*(*r*)) at each node *r* for all the central nodes of the *in silico* model and in the case of the central source-detector pair:

(8) $$e(r)=\left|\frac{v(r)-v_{\text{ref}}(r)}{v_{\text{ref}}(r)}\right|*100$$

where *v* is the Jacobian value and *v*_ref_ is the high photon count reference Jacobian value at the specific node. The nodes employed to calculate the error are highlighted in Figure 9a.

The high photon count reference Jacobian was computed using 10^10^ photons per optode. The error was computed for the Jacobian at the time corresponding to the 25% rising gate of the TPSF. A summary of the *e*(*r*) calculated for this configuration and for different photon packets is provided in Table 5.

In all cases, except for 10^6^ photons, *e*(*r*) was similar between initial and rescaled meshes/Jacobians. Note that repeating 10^10^ photons simulations on the initial mesh led to *e*(*r*)~2. Overall, 10^9^ produced the least *e*(*r*) with both field metric performing well, though they had 32% (ΣJacobian) and 34% (Attenuation Field) less nodes. The reduction in nodes and elements is mainly achieved in the central part of the mesh where the elements of volume are less visited by photons. The coarsening of the mesh in this region leads to improved statistics as seen in *e*(*r*) for early gates (note that *e*(*r*) is the average error as computed by the mean of the error at each node). In the case of 10^8^ photons, which is the typical number of photons used successfully for preclinical studies [14], the coarsening leads to a 14% reduction in error *e*(*r*). This suggests that mesh optimization as described herein can lead to reduction of the size of photon packets simulated and reduction in nodes/elements of volume for a more tractable inverse problem.

Additionally, we estimated the computational efficiency of the analytically rescaled Jacobian compared to MC re-computed Jacobians at each iterations. Time-resolved MC Jacobians were computed on 64 nodes of the CCNI’s Blue Gene/Q system at RPI whereas Jacobian rescaling was performed on a personal computer (i7-4930K Six-Core 3.40 GHz 12 MB Intel Smart Cache LGA2011, 64 GB DDR3/1600MHz memory) using in-house Matlab codes. To provide a meaningful comparison in terms of application, the simulations were performed on a 3D mesh for whole-body small animal imaging. The small animal was discretized in 15,581 nodes and 92,713 elements. 60 wide-field sources and 96 points detectors were employed [23]. To compute the time-resolved Jacobian, 210 min were necessary whereas the analytical rescaling took less than 15 min. Hence, for the 17 iterations it took to achieve convergence with the Attenuation field, the analytical rescaling approach was achieved within 7% of the time required to compute the MC sequential computations. Note that the analytical rescaling code was not optimized for parallel computing and that we typically use up to 1024 nodes on CCNI.

## 4. Conclusions

Mesh-based Monte Carlo techniques are relatively recent developments that promise improved computational efficiency for optical tomography. However, these techniques are not currently benefitting from mesh optimization techniques. Herein, we tested different solution fields with the goal of optimizing the forward model for computational efficiency. We found that the Σ*Jacobian* field and Attenuation field (*Att.*) field produced robust optimized meshes for preclinical applications. Moreover, we demonstrated that these solution fields maintained the accuracy of the forward model, especially for challenging data types such as early gates. Overall, the mesh optimization methodology described herein may be an attractive solution for computing initial meshes based on the attenuation field and/or rescaling mesh MC Jacobians for non-linear implementations in a computationally efficient framework.

## Figures and Tables

**Table 1:** Mesh characteristics at convergence under various size factor ranges.

Size Factor	Iterations	Nodes	Elements	Max. Elem. Vol. (mm^3^)
Initial	--	2396	7574	0.08
1.15	17	1332	3904	0.51
1.25	21	777	2200	1.49
1.35	20	612	1615	2.97

**Table 2:** Mesh characteristics at convergence under different solution fields.

Field	Iterations	Nodes	Elements	Max. Elem. Vol. (mm^3^)
ΣJacobian	21	777	2200	1.49
Log(ΣJacobian)	12	1487	4335	0.25
Normalized ΣJ	21	1053	3074	0.48
Curvature	15	447	1105	1.55
Log(Curv.)	20	668	1651	1.37

**Table 3:** Mesh characteristics at convergence under different solution fields.

Field	Iterations	Nodes	Elements	Max. Elem. Vol. (mm^3^)
ΣJacobian	2	1614	4872	0.26
Log(ΣJacobian)	5	1611	4792	0.25
Normalized ΣJ	5	1457	4334	0.25
Curvature	2	1136	3304	0.31
Log(Curv.)	2	1608	4846	0.25

**Table 4:** Mesh characteristics at convergence under different solution fields.

Field	Iterations	Nodes	Elements	Max. Elem. Vol. (mm^3^)
ΣJacobian	21	777	2200	1.49
Dist.	11	1675	5166	0.28
Att.	17	699	1999	1.80

**Table 5:** Errors in the forward model central nodes before and after Σ*Jacobian* mesh optimization.

Photons	Initial TG	Final TG Σ*Jacobian*	Final TG *Att.*
10^9^	10.84%	11.71%	13.99%
10^8^	36.83%	31.39%	35.55%
10^7^	61.01%	60.13%	58.57%
10^6^	86.71%	90.92%	151.43%
